# Supplementary material for: Prediction of RNA–protein interactions using a nucleotide language model
Source: Bioinform Adv. 2022 Apr 7;2(1):vbac023. doi: 10.1093/bioadv/vbac023 (PMC9710633; doi:10.1093/bioadv/vbac023)
Supplement: vbac023_Supplementary_Data [file vbac023_supplementary_data.zip › BERT_RBP_revision_suppl.pdf]

Supplementary materials for "Prediction of RNA-protein interactions using a nucleotide language model"

Keisuke Yamada and Michiaki Hamada

List of Tables

1 Hyperparamaters used during the training of BERT models 2

List of Figures

1 Detailed comparison of BERT-RBP's performance 3
2 Cross-validation performance 3
3 Comparison of different k-mer models 4
4 The performance of modified BERT 4
5 Shift of specialization 8
6 Extracted RBP binding motifs 8

Supplementary Table S 1: Hyperparamaters used during the training of BERT models

Hyperparameter	BERT-baseline	BERT-RBP	BERT-RBP(with CNN)	BERT-RBP(CLS average)
Batch size	64	64	64	64
Learning rate	6e-5	2e-4	2e-4	2e-4
Epoch	5	3	3	3
Warmup rate	0.1	0.1	0.1	0.1
Dropout probability	0.01	0.1	0.01	0.01
Weight decay rate	0.01	0.01	0.01	0.01

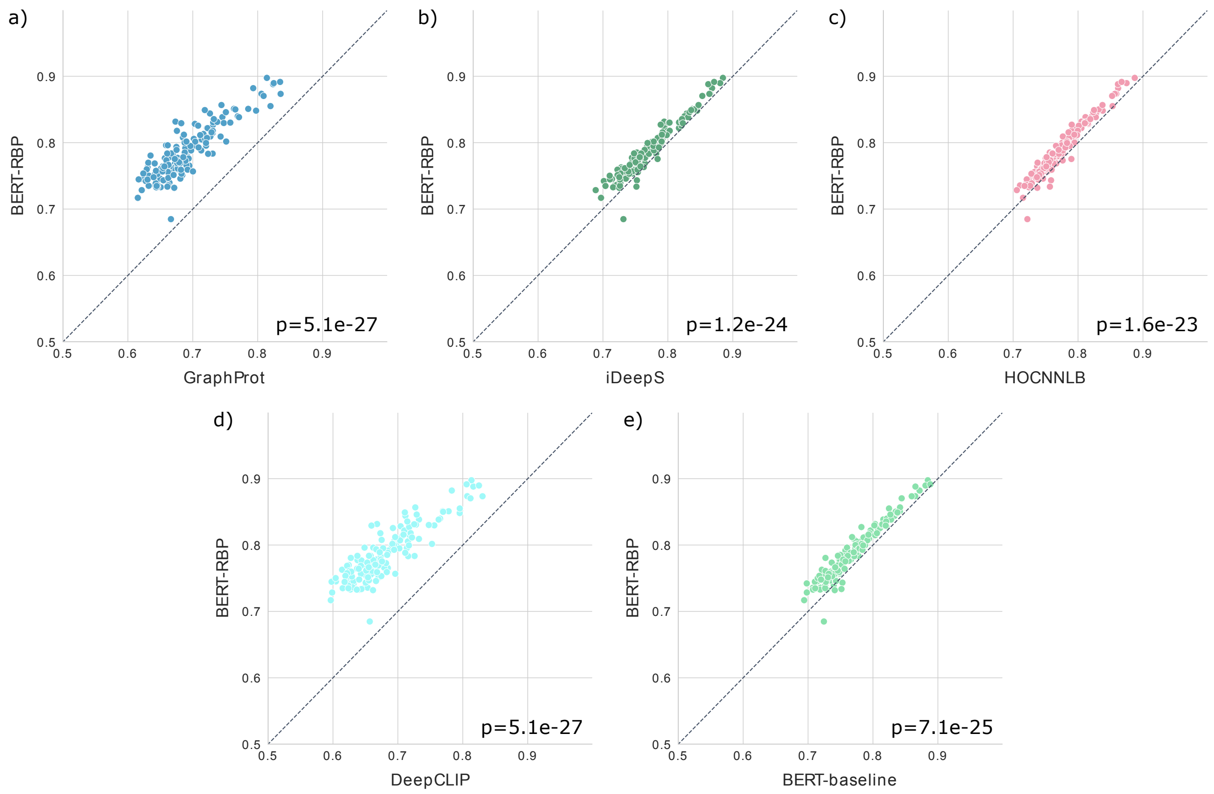

Supplementary Figure S 1: Detailed comparison of BERT-RBP's performance against a) GraphProt (Maticzka *et al.*, 2014), b) iDeepS (Pan *et al.*, 2018), c) HOCNNLB (Zhang *et al.*, 2019), d) DeepCLIP (Grønning *et al.*, 2020), or e) BERT-baseline by the measure of AUROC. Each dot represents the AUROC scores of BERT-RBP and the corresponding baseline model trained using the same RBP dataset. The diagonal dashed line indicates where the performances of the two models are identical. p-values were calculated using Wilcoxon's signed-rank test.

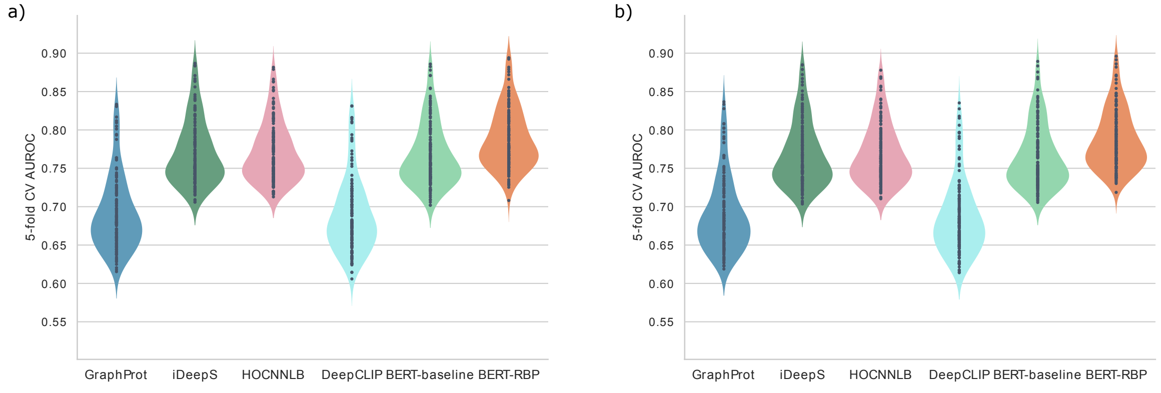

Supplementary Figure S 2: Cross-validation performance was measured using area under the receiver operating characteristic curve (AUROC) scores for BERT-RBP and four baseline models over 154 RBP datasets. Each violin plot shows the performance of each model, and each dot within each violin plot represents the AUROC score for a single RBP dataset. Training and evaluation datasets were used for a), and low sequence-similarity datasets were used for b).

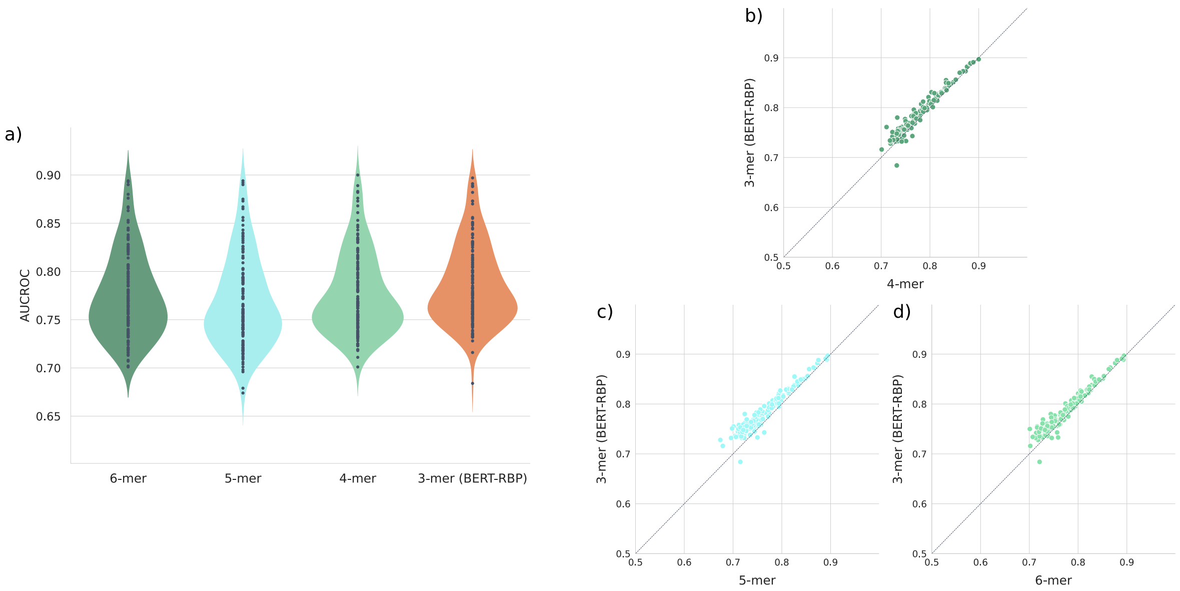

Supplementary Figure S 3: Comparison of different k-mer models. a) Area under the receiver operating characteristic curve (AUROC) scores of models pre-trained and fine-tuned with different k-mer representations in over 154 RNA-binding protein (RBP) datasets. Each violin plot shows the performance of each model, and each dot within the violin plot represents the AUROC score for a single RBP dataset. b-d) A detailed comparison of BERT-RBP's performance against b) 6-mer, c) 5-mer, or d) 4-mer models by AUROC measurement. Each dot represents the AUROC scores of BERT-RBP and the target model trained using the same RBP dataset. The diagonal dashed line indicates that the performances of the two models are identical.

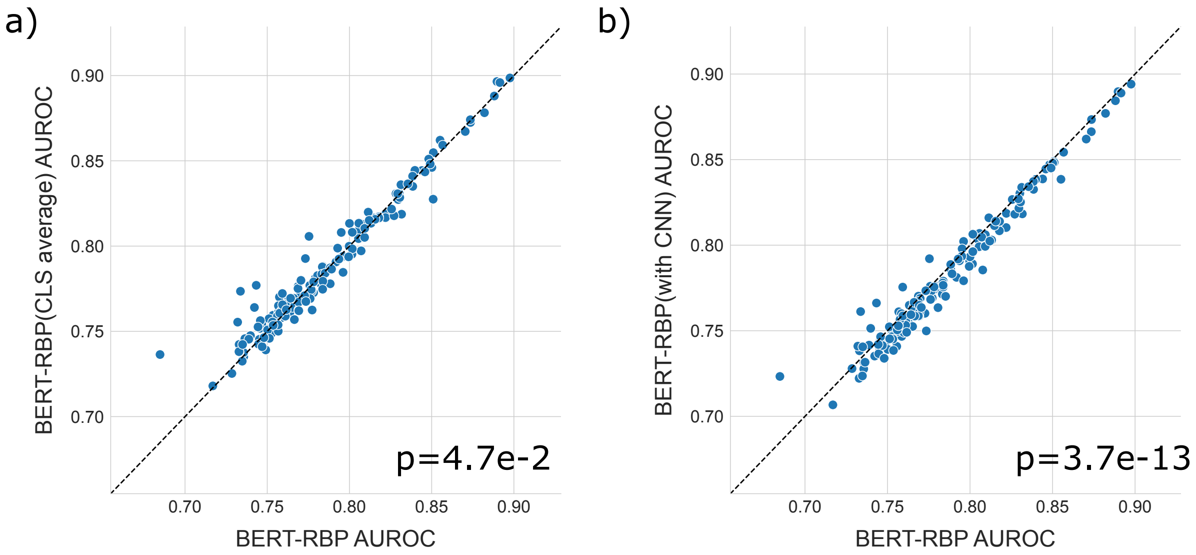

Supplementary Figure S 4: Detailed comparison of BERT-RBP's performance against a) BERT-RBP with a convolution layer after the final layer and b) BERT-RBP that uses the weighted average of CLS tokens from all hidden layers. Each dot represents the AUROC scores of BERT-RBP and the target model trained using the same RBP dataset. The diagonal dashed line indicates that the performances of the two models are identical. p-values were calculated using Wilcoxon's signed-rank test.

References

Bailey, T. L. (2021). STREME: accurate and versatile sequence motif discovery. *Bioinformatics*, **37**(18), 2834–2840.

Feng, H. *et al.* (2019). Modeling RNA-Binding protein specificity in vivo by precisely registering Protein-RNA crosslink sites. *Molecular cell*, **74**(6), 1189–1204.e6.

Grønning, A. G. B. *et al.* (2020). DeepCLIP: predicting the effect of mutations on protein-RNA binding with deep learning. *Nucleic acids research*, **48**(13), 7099–7118.

Ji, Y. *et al.* (2021). DNABERT: pre-trained Bidirectional Encoder Representations from Transformers model for DNA-language in genome. *Bioinformatics*, **37**(15), 2112–2120.

Maticzka, D. *et al.* (2014). GraphProt: modeling binding preferences of RNA-binding proteins. *Genome Biol.*, **15**(1), R17.

Pan, X. *et al.* (2018). Prediction of RNA-protein sequence and structure binding preferences using deep convolutional and recurrent neural networks. *BMC genomics*, **19**(1), 511.

Zhang, S.-W. *et al.* (2019). Prediction of the RBP binding sites on lncRNAs using the high-order nucleotide encoding convolutional neural network. *Analytical biochemistry*, **583**, 113364.

EWSR1

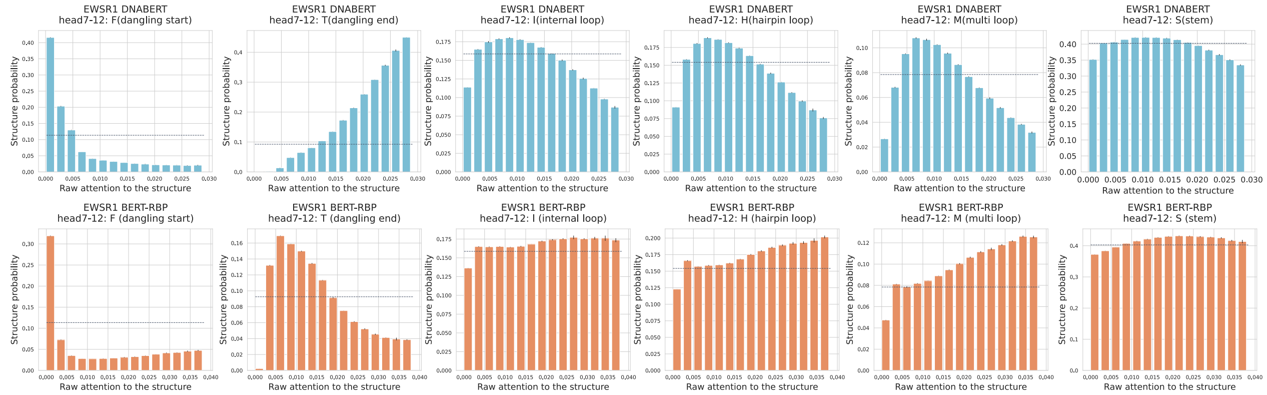

FUS

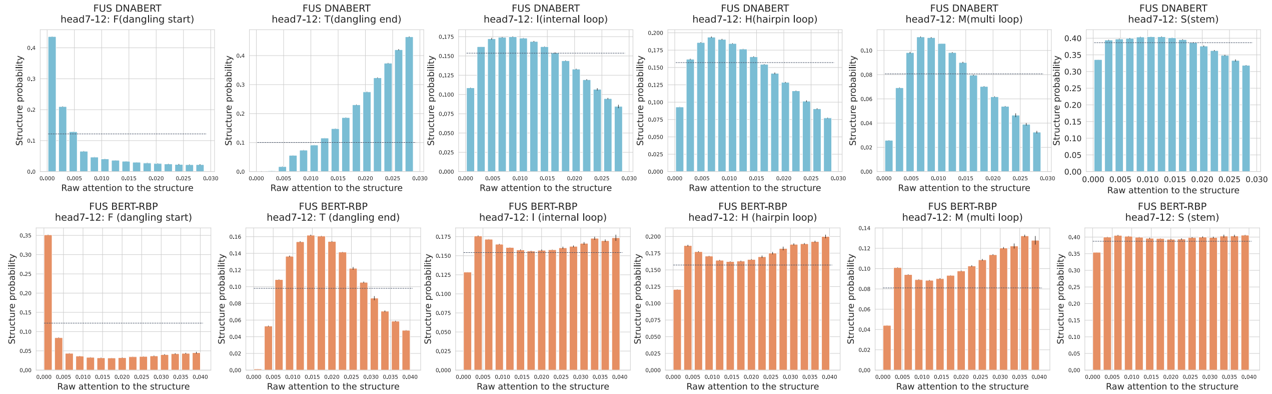

HNRNPK

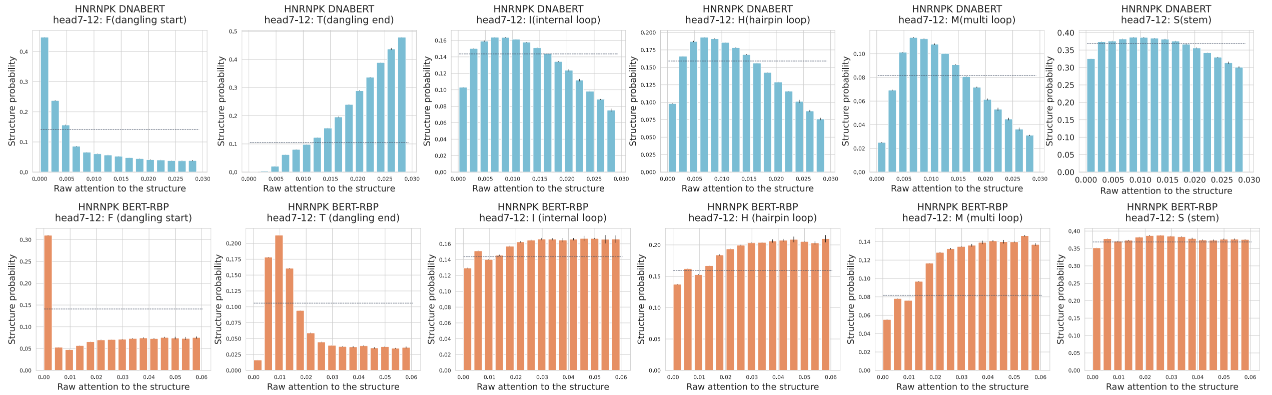

RBM22

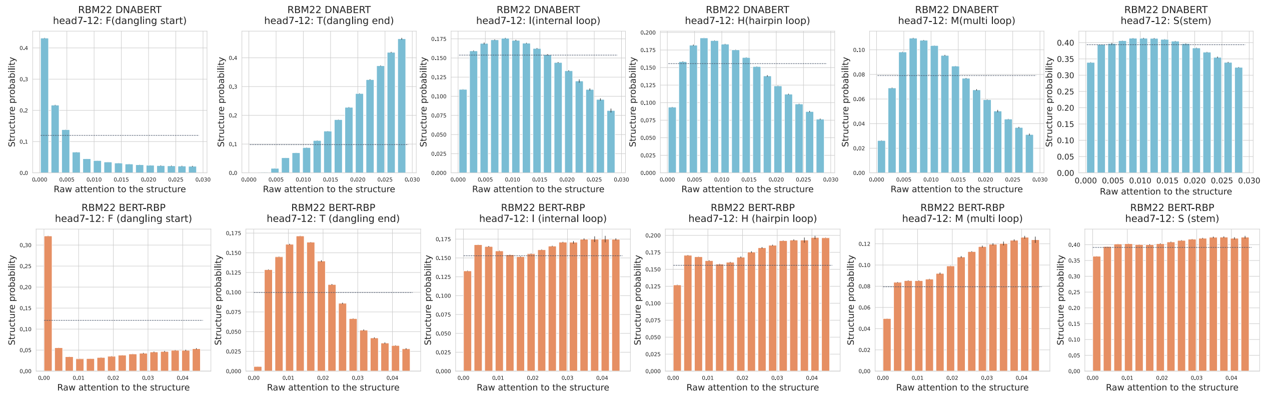

SRSF1

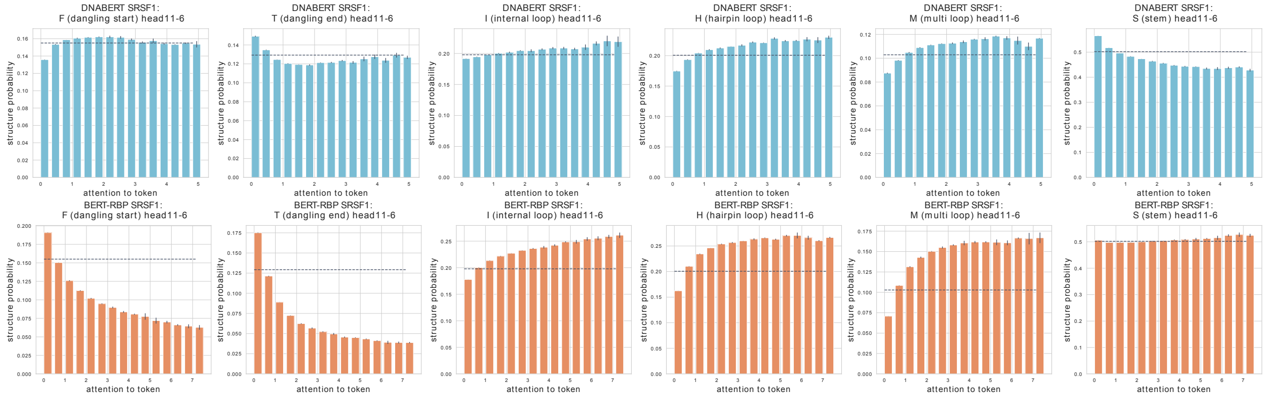

SRSF9

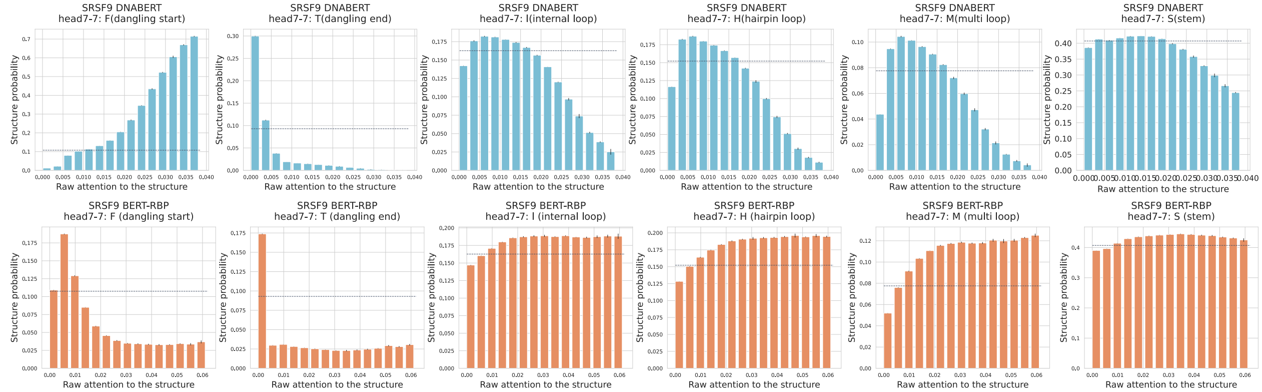

TAF15

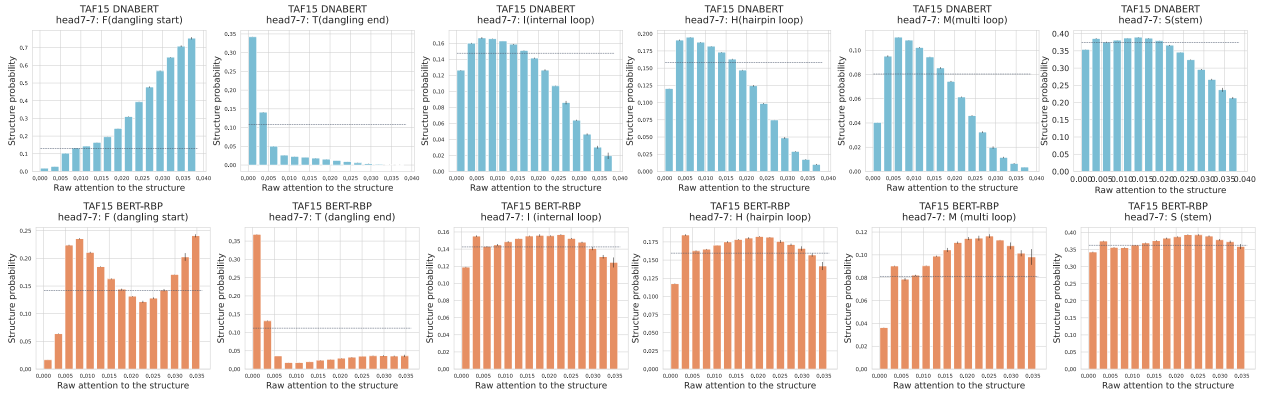

TIA1

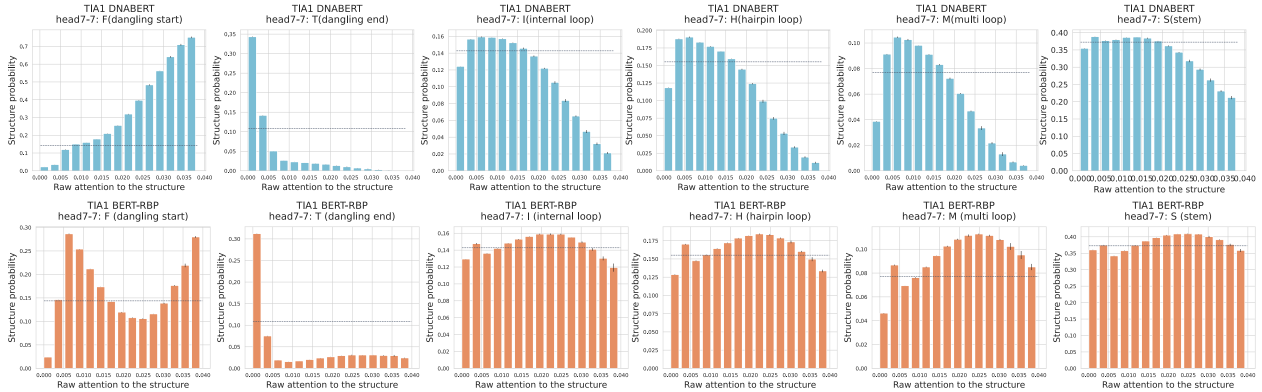

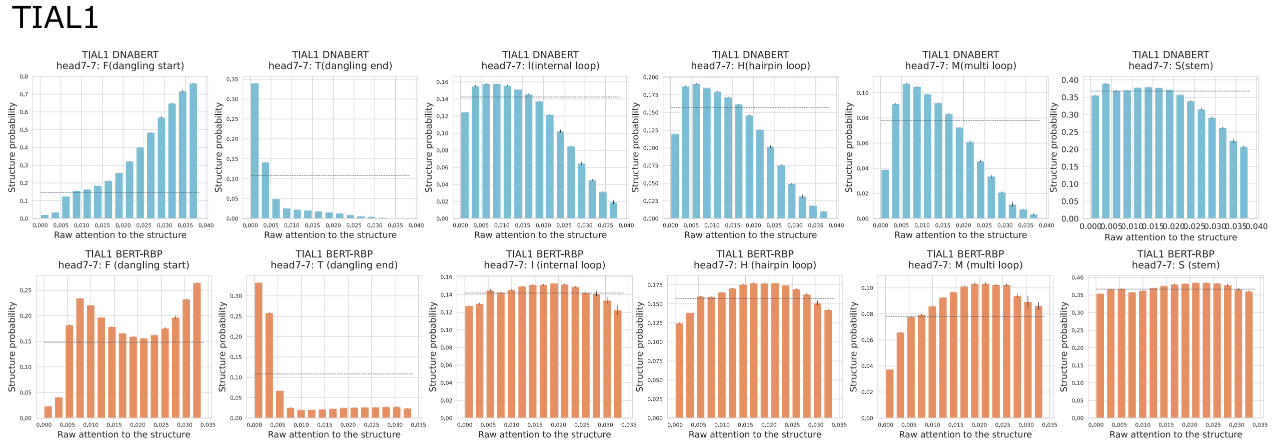

Supplementary Figure S 5: The detailed results of correlation analysis for nine RNA-binding proteins (RBPs) that showed the shift of specialization. A detailed analysis was conducted to inspect correlations between the raw attention to the RNA secondary structure type and the token probability to incorporate the structure at the selected head. For each BERT-RBP, the attention head with the highest attention ratio to the structure was chosen (bottom, orange), and the head of DNABERT (Ji *et al.*, 2021) at the same position was used (top, blue). The horizontal dashed lines represent the background probability of the structure label within each RBP non-training dataset. Error bars represent means ± standard deviations among three subsets randomly split from the original non-training set.

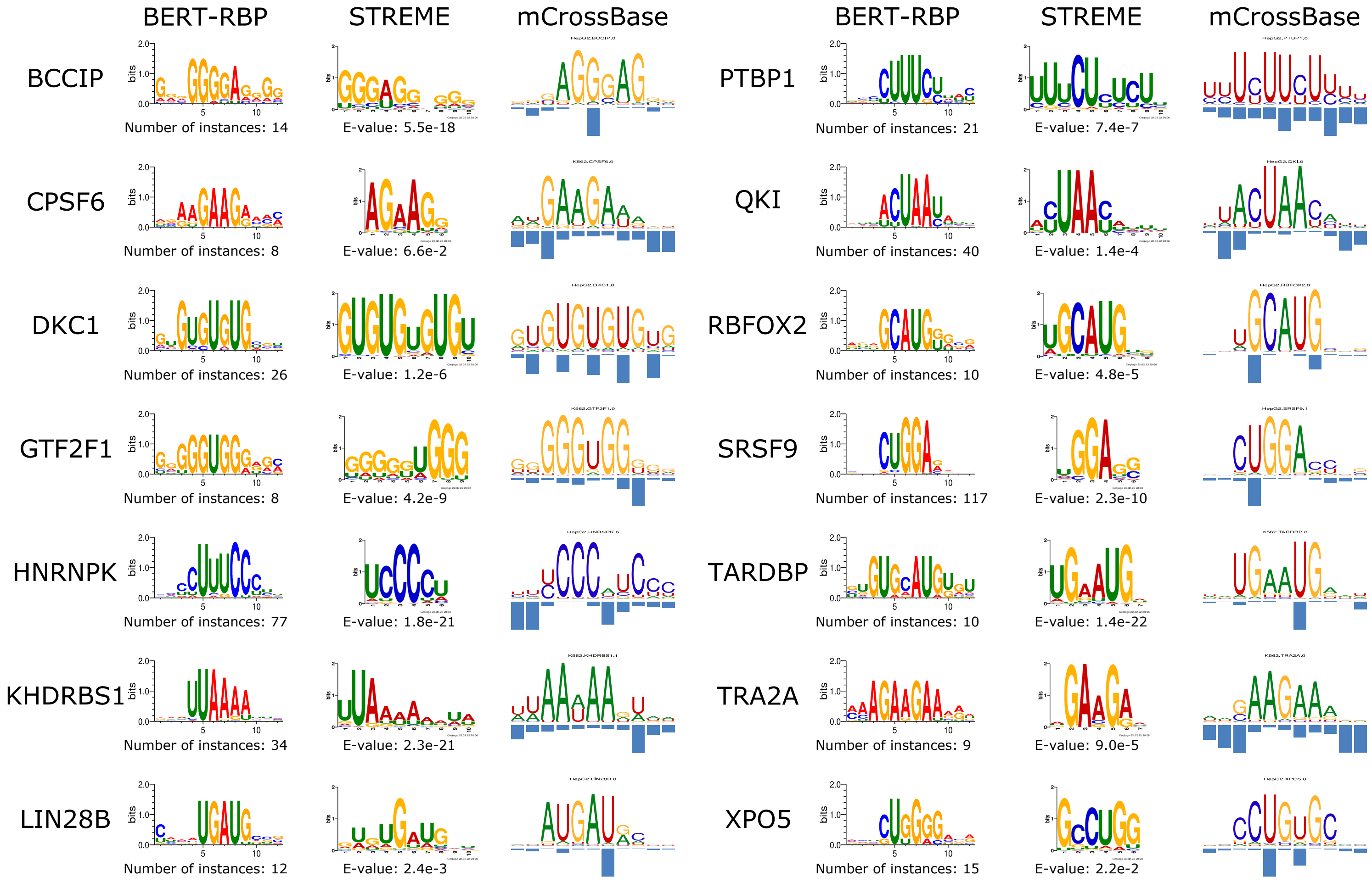

Supplementary Figure S 6: Exemplary motifs extracted from BERT-RBP that agree to motifs detected by STREME (Bailey, 2021) and motifs downloaded from mCrossBase (Feng *et al.*, 2019).
